# A qualitative interview study on the positive well-being of medical school faculty in their teaching role: job demands, job resources and role interaction

**DOI:** 10.1186/s13104-015-1393-4

**Published:** 2015-09-02

**Authors:** J. W. van den Berg, C. P. M. Verberg, J. J. Berkhout, M. J. M. H. Lombarts, A. J. J. A. Scherpbier, A. D. C. Jaarsma

**Affiliations:** Center for Evidence-Based Education, Academic Medical Center-University of Amsterdam, Meibergdreef 15, Room J1A-138, 1105 AZ Amsterdam, The Netherlands; Faculty of Health, Medicine and Life Sciences, Maastricht University, Maastricht, The Netherlands; Center for Research and Innovation in Medical Education, University Medical Center Groningen, University of Groningen, Groningen, The Netherlands

**Keywords:** Work engagement, Job demands and resources, Work environment, Role interaction, Medical school faculty

## Abstract

**Background:**

Attention for the well-being of medical school faculty is not only important for the prevention of attrition and burnout, but may also boost performance in their tasks in medical education. Positive well-being can be conceptualized as work engagement and this is associated with increased performance. In this study we explore how demands and resources from different tasks affect work engagement specifically for education.

**Methods:**

Between June and September 2013, we conducted a multisite semi-structured interview study with a diverse group of medical school faculty and used an open-coding strategy within the Work Engagement Model on the transcribed interviews.

**Results:**

We interviewed 16 faculty members whose teaching experience ranged from 7 to 38 years and whose professional tasks ranged from being solely an educator to being a physician, researcher, educator and administrator simultaneously. All participants were clear on the perceived demands and resources, although similar aspects of the work environment could be perceived oppositely between participants. Overarching themes were perceptions related to the organization or department, often described as a general and long-term effect and perceptions directly related to a task, often described as a direct and short-term effect on well-being. Furthermore, the demands and resources as resultant of fulfilling multiple tasks were described clearly by participants.

**Conclusions:**

The ambiguous nature of the work environment in terms of demands and resources requires an individualized approach to supporting work engagement. Furthermore, faculty members perceive many resources from fulfilling multiple tasks in relation to their tasks in education. Faculty developers and administrators alike could use these findings to apply the concept of work engagement to their daily support of faculty in medical education.

## Background

There is plenty of reason to be concerned about the well-being of medical school faculty: compared to the general population, burnout prevalence has been shown higher in physicians [1, 2], who make up a large part of medical school staff. As indicators for burnout have a high prevalence among physicians across Northern America and Europe alike [3–7], this concern seems to transcend local and even national boundaries.

Research specifically focused on medical school faculty [8–10] and professors in medical education [11] has shown similarly high burnout rates indicating it is equally a concern specific to academic medicine. This is concerning because, among other things, faculty burnout is associated with intent to leave academic medicine [8]. Research on intent to leave and attrition shows it is prevalent among faculty [12–17]. It appears most prevalent in early stages of physicians’ careers [18–20], academic careers [16] and among younger faculty [11]. It has been suggested that physicians’ well-being should be a quality indicator for healthcare [21] and a similar call could be made for the well-being of medical school faculty. However, solely preventing burnout does not necessarily equal achieving the positive spectrum of well-being. As has been noted by Shanafelt et al. [22], it is not merely the absence of distress that is required for positive well-being.

The positive antithesis to burnout is work engagement. Work engagement is defined as an active, positive, work related state of being characterized by vigor, dedication and absorption [23]. These three dimensions of work engagement enable those with higher levels of work engagement to perform better, because of higher energy levels and mental resilience, being involved in one’s work and experiencing significance, enthusiasm and challenge and being concentrated and engrossed in work [23]. Several concepts are related to but different from work engagement [24]. One of these is job satisfaction, which is more concerned with one’s affect towards work, whereas work engagement refers to one’s mood at work. Also, satisfaction is generally a passive state, whereas engagement is an active state of being. Consequentially, work engagement more strongly associates with performance than does job satisfaction [25]. A second concept related to work engagement is workaholism, because both involve being immersed in one’s work. However, for work engagement this is driven by an intrinsic motivation, whereas for workaholism this is driven by a compulsory drive. Consequentially, workaholism is associated with a higher burnout risk and work engagement protects against burnout [26].

Being engaged to work not only protects against burnout, it is also associated with better overall health and increased performance [23, 27, 28], i.e. residents report fewer medical errors [29] and clinical teachers being better supervisors [30]. As such, supporting the work engagement of medical school faculty may be an important lead for faculty developers, human resource management (HRM) and boards alike both for increasing the quality of their education as well as in taking care of their staff.

**Fig. 1:**
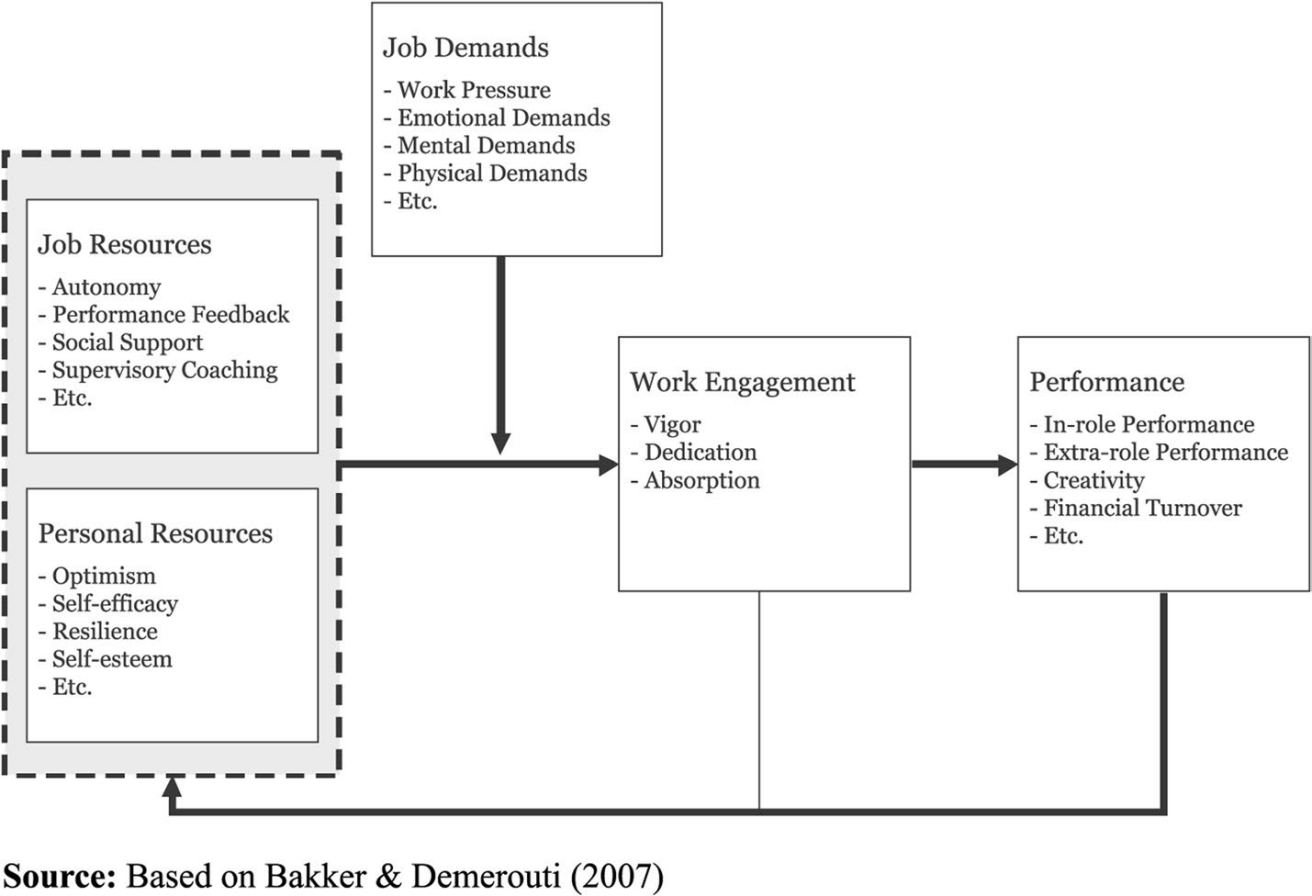
Work engagement model, as published in [32] based on [33], graphically representing interaction between resources, demands, work engagement and performance

However, in order to support work engagement of medical school faculty, it is necessary to understand what drives work engagement for teaching in medical education. In general, work engagement is driven by job resources and personal resources. Job resources are the physical, social or organizational aspects of the job that reduce job demands, are functional in achieving work goals and stimulate personal growth. Similarly, personal resources are ‘aspects of the self that are generally linked to resiliency and refer to the individuals’ sense of their ability to control and impact upon their environment successfully’ [31]. The influence of these resources on work engagement is mediated by the job demands, those job related aspects that require sustained physical, cognitive or emotional effort or skills to overcome [23, 27, 28]. For example, job resources include performance feedback and social support and job demands include perceived work pressure, due to time constraints or work load. These relations are visually represented in the Work Engagement model (Fig. 1) as published in [32] based on [33], which builds upon the Job Demands-Resources model [33].

The Job Demands-Resources model can be used to describe how job and personal characteristics interact and how this affects well-being. An important difference between the application of this model on work engagement and its application on burnout is that perceived demands are directly related to burnout, whereas for work engagement demands actually increase the positive effect of resources [34].

For the medical education context specifically, Scheepers et al. [30] found different levels of work engagement in terms of clinical teaching and patient care for academic medicine in particular, while one other study has shown that the level of work engagement for education decreases when faculty have tasks in research and clinical care in addition to education [35]. This suggests that the demands and resources differ between tasks for faculty within the same institution and work engagement should be studied for education specifically.

Previous research into work engagement has related some motivational resources directly to work engagement for education [35] and both full-time and part-time United States’ medical school faculty are satisfied with the resources provided [36]. However, a comprehensive study into *how* different tasks relate to work engagement for education and where both resources and demands are included in the study, has so far not been conducted. In order to better understand this process, this study addresses two research questions: (1) how do medical school faculty describe their work environment in terms of demands and resources in relation to their teaching tasks? and specifically (2) how do medical school faculty describe the interaction between other tasks and roles in relation to their teaching task?

To answer these questions we conducted a multisite semi-structured interview study with a diverse group of medical school faculty and used an open coding strategy within the Work Engagement Model on the transcribed interviews.

## Methods

### Study design

Our approach to the research questions was based on the interpretivist paradigm, because we acknowledged reality is subjective and changing and that no ultimate truth exists [37]. We followed this paradigm as the roles faculty fulfil may be perceived and experienced differently by individuals. Furthermore, the work environment of medical faculty is heavily influenced by rapid changes in technology and knowledge. As our focus was on understanding how demands and resources interact, diverse interpretations of these aspects of the work environment needed to be collected. We conducted semi-structured, individual interviews to allow in-depth discussion in an enclosed environment in which participants felt safe to talk about their perceived resources and demands [38]. Our sampling was purposeful and iterative; the analysis was done iteratively and based on sensitizing concepts. This will be detailed below [39].

### Setting and participants

Our study was conducted at two university hospitals in the Netherlands. Both offer medical education along the entire continuum. In Dutch university hospitals the majority of faculty members are expected to provide patient care, participate in education and also produce scientific output. Furthermore individual faculty members are often part of boards, committees or otherwise participating in the organization of education, research or patient care. The background of faculty is in both medicine and basic sciences. Given our desire to collect diverse interpretations of the work engagement concept, we purposefully sampled our participants for maximum variation [38, 40] to ensure a wide range of backgrounds and individual characteristics. We based our sampling primarily on tasks other than teaching: in patient care, administration, education and research [41]. We further sampled on background and specialty to ensure the sample spanned different departments. For this sampling, we defined tasks in education as broader than teaching, including the design, development or management of teaching at a departmental or institutional level, i.e. a course director or clerkship director. Furthermore, we aimed to include teachers with a substantial teaching role, which we defined as teaching at least on a monthly basis on average and to include representation from both university hospitals.

For logistical reasons, invitations were sent in batches of three to five emails, so interviews and subsequent analysis could be planned in advance. Nine faculty were unable to participate owing to their schedule or did not respond. In such cases, another faculty member with a similar profile was invited. Invitations stopped when saturation was reached. Interviews were conducted at location of choice of teacher and all interviews were held at faculty’s main working location.

### Data collection

The interview guide and topic list were designed based on the Work Engagement Model [23]. Topics included perceived resources, perceived demands and the perceived effect of fulfilling multiple roles on teaching. Before formal data collection started, one pilot interview was conducted and discussed among the authors to ensure appropriate interviewing techniques were used. One researcher (JvdB, a PhD-student, medical doctor and first author of this study) conducted all semi-structured interviews between June and September 2013, which lasted between 40 and 68 min.

Participants were first asked to provide an example of a positive experience in teaching, as an icebreaker question. Second, our definition of work engagement based on the literature was elaborated on during the interview between the opening question and first topic to ensure the theoretic background of the interview was clear. We felt this was necessary because the pilot-interview showed that the day-to-day meaning of the Dutch denotation for work engagement (‘bevlogenheid’) could imply a passive and uninfluential form of being inspired. The interviewer described work engagement as an active, positive work related state characterized by vigor, dedication and absorption. The first topic was introduced by asking the participants how they were enabled to be engaged with teaching in their daily work. The formulation of this question was open-ended and allowed participants to report anything that came to mind. Participants were asked to elaborate on their statements and to give examples where possible. If necessary, the interviewer introduced the topic of resources, demands and role interaction with an open question.

### Data analysis

Each interview was audio-recorded, transcribed verbatim and anonymized; participants were given a pseudonym instead. A summary was sent to the participants for respondent verification, all participants verified this summary. MaxQDA Version 11 (Verbi GmbH, Berlin, Germany) was used to support analysis. Analysis started directly after the first interview and continued simultaneously with data collection.

The topics chosen for our interviews based on the Work Engagement Model also served as sensitizing concepts for our analysis, whereby the concepts of job resources, job demands and role-interaction were initially regarded as main themes. Associated keywords for a resource could indicate positive emotions or expressions such as “makes my job easier”, associated keywords for a demand could indicate negative emotions or expressions such as “it takes away energy”. We also made notes if such comments were made in relation to one role or if these affected other roles. An open-coding strategy was used whereby descriptive codes were attached to participant quotations, staying close to participant wording. One quotation could contain multiple codes. During analysis, codes and results were discussed among the other authors regularly to further refine coding and consider emerging themes. Specifically, the third interview was independently coded by a second researcher (CV) and through discussion of emergent differences it was found that the initial sensitizing concepts did not cover all participant quotations. We again reviewed our codes using work engagement literature and decided to add as sensitizing concepts performance feedback and resources and demands on a personal level as sensitizing concept. More specifically, we included in the template whether a resource or demand was attributable to the work or the participant, using the same keywords as before. The ninth interview was again independently coded by the second researcher (CV) and differences were discussed. At this point the codes remained unaltered but an alternative arrangement of themes was chosen to better fit the data. In particular, the *origin* of the resources and demands were set as main themes and within these themes a distinction between perceived resources and demands was made, instead of placing ‘demands’ and ‘resources’ as main themes. The thirteenth interview was independently coded by a third researcher (JB) and after discussion of emergent differences it was concluded that the coding structure did not need further adaptation. In the remaining interviews no new themes emerged and saturation was considered to have been reached. After each revision of the themes and codes, all previous interviews were revisited and analyzed with the new coding structure.

### Ethical considerations

Approval of the Netherlands Association for Medical Education-Ethical Review Board was obtained under dossier number 234. Participants received a gift certificate after the interview, but were not notified of this in the invitational email to further ensure voluntary participation. Confidentiality was guaranteed in the invitation and confirmed at the start of the interview.

## Results

### Participants

The 16 participants included 7 (44 %) practicing physicians, 3 (19 %) teachers with a degree in medicine who no longer practised and 6 (38 %) basic scientists. Most participants fulfilled two additional roles. 1 (6 %) physician fulfilled all possible roles, being an administrator, educator and researcher in addition to being a teacher and physician. Teaching intensity ranged from 10 h of lecturing per year to daily supervision of clerks and residents. Table 1 lists participant details.

**Table 1:** Participant details

	# (%)	Teacher-Administrator	Teacher-Educator	Teacher-Researcher	Teacher-Clinician
Physicians	7 (44)	1	4	5	7
Non-practicing physician (MD degree)	3 (19)	2	3	2	0
Basic scientists	6 (38)	1	3	5	n/a

	Yearly hours involved in education	Years of experience	Male/female (%)	PhD/non-PhD (%)	
Range or ratio	10–1000+	7–38	11 (69 %)/5 (31 %)	12 (75 %)/4 (25 %)	

### Identified themes

During the interviews, the participants were very clear about what they perceive as influencing their work as a teacher and about how their work environment affects their teaching role. They were generally able to elaborate clearly and consciously on the causes and effects of these influences. Subsequently, many separate influences and perceptions could be categorized as either demand or resources, which we could categorize in five main themes: (1) *organization and department related demands and resources*; (2) *task*-*related and*; (3) *personal demands and resources*; (4) *role interaction* and (5) *teacher actions*. Within these themes, elements could not unambiguously be identified as a resource or demand, this labeling was found to be very personal and thus varied between participants. A sixth theme, *processes*, highlights *how* the demands and resources affect the teacher and *how* these demands and resources could be connected and may interact.. We will first describe these processes, then describe how demands and resources differ at the organizational level, task level and personal level followed by their descriptions of the role interaction. We end with the actions participants described as a result of the perceived demands and resources.

In the presentation of our results, we have aimed to use representative quotations from different participants. We did not deem it necessary to present a quotation from every participant but selected the most illustrative ones. In addition, one example from Agnes, a clinician and educator, on marking multiple choice questions was illustrative from several perspectives and will therefore be referenced more than once.

Table 2 concisely provides an overview of the five main themes along with practical examples for each theme as provided by one participant versus a contrasting one provided by another participant. This table does not include the “Processes” theme.

**Table 2:** Representative resources and demands

	Resource versus demand
Organisational level	
Colleagues	Experienced colleagues versus those poor in cooperation
Support	Faculty development versus no financial compensation for teaching
Curriculum	Academic freedom versus little appreciation of specialism in curriculum
Systems and policy	Career opportunities versus poorly implemented educational awards
Culture	Active educational mission versus an unappreciative top-down approach
Task level	
Design and preparation of a session	Being assigned learning goals for the session versus having to use someone else’s slides
Within the teaching session	Small group session versus afternoon lecture
Subsequent examination and assessment	Using exam results to provide personalized feedback versus having to provide negative feedback
Student interaction	Curious students with clever questions versus disruptive students who show up late
Personal level	
	Need to perform versus perfectionism
Role interaction	
	Invigorated by successes versus scheduling conflicts
Teacher actions	
Altering their work environment	Ignoring quality assurance evaluations
Altering their teaching task	Do more than meeting the learning goals or ignore set rules
Altering their other tasks	Stop doing biomedical research or start doing education research

### Processes

The participants described elements of their work environment as resource, e.g., being helpful or giving energy, and as demand, e.g., being frustrating or costing energy. In addition, in their descriptions of these elements, their elaborations also gave insight in *how* and *why* these elements had an effect on their well-being in the teacher role.

To illustrate this, we first provide a short citation from Agnes. Speaking of no longer having to mark multiple choice questions herself, she began her explanation by saying “*Lately*, *err.. actually for several years now*”. It was actually one of the first examples she gave in the interview, so this indicates that it was an important change for her. Furthermore, this seemingly small change in her teaching task brought relief for a longer duration in time, in addition to the immediate effect it had when the change was introduced, as apparently this change was introduced years ago but still brought relief in addition, it is something tangible.

A contrasting example was given by Edward, a clinician, researcher and educator: “*For example*, *when you*’*ve just heard an article has been accepted in a high quality journal*, *then your lectures will be better*, *it gives a boost.*”

Edward clearly perceived such a success as having an immediate and positive effect on his subsequent teaching, but also noted this was a relatively short-term effect. This perceived resource also serves as an example of something more abstract compared to the previous quotation and less easily realized, but rather something which happens by chance.

The last example that indicates how resources and demands may affect teachers in general was given by Leonard, a clinician and researcher, who commented on the organizational policy towards low-performing students: “*There*’*s an atmosphere in which students are required to perform well and are completely motivated and energized from the start.* […] *And when they fail one year*, *they*’*re immediately branded as problem*-*case.* […] *That makes me mad.*” Leonard too wanted his students to try their best and perform well, but he had compassion for those who would fail one exam. He believed these students deserved a second chance. This organizational policy did not affect Leonard directly, but rather it affected him indirectly through its impact on students. Furthermore, this aspect did not affect Leonard continuously, but would come to the foreground only now and then. This illustrates the lagged and indirect effect resources and demands may have on faculty.

These descriptions from participants of *how* and *why* resources and demands affected them varied in the immediacy of the effect, duration of the effect and concreteness of the influence.

### Organization and department related demands and resources

A large part of the demands and resources our participants described were related to the institution they worked at, at times specifically including the department. Within this theme we distinguished five institutional and departmental elements: were culture, systems, policies, support and colleagues. In addition participants also described elements of the curriculum they taught in, An example of a demand perceived by Bert, a researcher, is: “*The educational awards for best teachers are alright*, *but I think the awards are really about how enjoyable the lecture is*, *how enjoyable the practical is*, *but that doesn*’*t make that person the best teacher.*” This example shows how the institution put effort in acknowledging excellent teaching, but did achieve that result for Bert as he described the educational awards as frustrating. To him these felt like an empty gesture and not a sign of education being taken seriously. Several other participants actually described that educational awards were, for them, a sign that education was being taken seriously and that they were being acknowledged by these teaching awards. This further highlighted the individuality of the perceived demands and resources. An example of how policy could also positively affect our participants related to what Irma, an educator, said: “*I am lucky to have a teaching appointment. Some colleagues have a research appointment* […] *and have to raise funds and fulfill educational tasks. I have the luxury to be appointed specifically for education.*”

The positive effects of appointing certain faculty for teaching specifically, rather than providing patient care or scientific research was twofold. Foremost, it meant she had protected time for teaching but second, this also showed to her and others that teaching was a viable career alternative.

Another example of how support can be given to teachers is through faculty development, which serves a dual function: it helps teachers obtain educational skills but can also be a sign in itself that education is taken seriously.

The demands and resources at this level were mostly described as a more general effect on their teaching tasks and these effects would generally affect the teacher over a longer period of time. They could be related to tangible elements, such as the policy of appointing someone for teaching specifically, but also to more abstract elements, such as the perceived recognition for teaching within a department.

### Task-related demands and resources

Several aspects of the work environment were described as directly related to the task of teaching.

Both the task and the work environment could directly influence the teaching session, but our participants also described influences on their preparation and on subsequent examination and assessment. An overarching element in the task-related demands and resources was student interaction. As a first example of an initiative which directly made a teaching task easier was given by Agnes when she said: “*Fortunately*, *we no longer have to mark multiple choice questions ourselves* […] *for several years now*, *which really makes me happy*”. This citation shows the immediate and specific effect of this initiative on this participant, in addition to the longer lasting effect reported before. A task that she considered as time-consuming and a chore, and therefore costing emotional effort to fulfill, had been made far less demanding.

Student interaction was described as energizing by all our participants, but perceptions varied about what elements of this aspect made this so. To some, it was rewarding to pass on their knowledge and to others it was akin to caring for patients and therefore provided a sense of fulfillment. On the other hand, as Henry, a clinician and researcher, nuanced: “*When* [*a student*] *says to me:* ‘*You can do what you want*, *but I*’*m not interested.*’ […] *That*’*s a bitter pill to swallow*”. Therefore, even though overall working with students is positive, certain students and aspects of teaching still required emotional or cognitive effort or skills to overcome.

Participants usually described these task-related demands and resources as having an immediate and short-term effect. In addition, the examples given were mostly tangible and also relatively easy to achieve.

### Personal demands and resources

Our participants spoke consciously of what part of themselves they bring into teaching. These demands and resources could be role specific, such as feeling competent, but could be more general as well when describing personality traits. Some teachers spoke of personal traits which were beneficial to their teaching but not limited to the teaching task, such as Gwen, a researcher and educator, “*I want to perform. I have aspirations*, *I want to move forward. And that fighting spirit or passion*, *or whatever you want to call it*, *that*’*s just within you.*”

Other teachers were also aware of personal traits which acted in a more detrimental way. One such example involved a feeling of insecurity when positive feedback was absent, which led to a perceived increased effort to perform well. Leonard: “*There*’*re people who are truly confident in themselves*, *all sorts of them. But for me*, *when nothing* [*positive*] *happens*, *I gradually become insecure*”.

Both these examples show personal demands and resources which effect isn’t limited to teaching, but the strength of the effect could be specific for teaching. These demands and resources encompass traits and characteristics as well as beliefs and preferences. Their role in work engagement is perceived as important by all participants.

### Role interaction

During most interviews there was no need to prompt participants to talk about role interaction as they would describe such occurrences naturally. The interviewer was prepared to ask participants about this topic to learn more about the role interaction. Most participants talked very positively about fulfilling different roles, albeit sometimes in an abstract manner, whereas more practical examples usually revolved around scheduling conflict. A positive example of role interaction was provided by Leonard, a clinician and educator: “*The feeling you are reckoned for your clinical work and research*, *makes you a different teacher*”.

Edward, a clinician and researcher, describes a typical night and day in which the different tasks literally conflict in his schedule. During his description of this day, he clarified that the scheduling conflict was not what bothered him but the continuing shift of focus from one task to another, while being somewhat sleep deprived, costs a lot of energy.: “*On the night from Monday to Tuesday*, *I had a night shift and then I get called a few times* […] *and then Tuesday morning we have the patient transfer at 8 and then at 9 you*’*re giving a lecture…*”

The more general and longer lasting effect of role interaction was the variety in tasks it provided whereas the direct and immediate effect was related to where roles overlap. This overlap could be in practical terms whereby skills from one role could be applicable to another, but also in scheduling terms whereby it increased work pressure.

In dealing with fulfilling multiple roles, we want to highlight one particular phenomenon described by Nico, researcher, administrator and educator: “*I don’t have any problems with* [*closing myself off*]*… As a figure of speech*, *if I were to have an unpleasant conversation with you now*, *then I can still happily get on with my other work. I can separate such things really well.*” He and other participants spoke of being able to close themselves off and being ‘in the moment’ when performing in their teacher role. We have singled out this citation as this may be an exceptionally important skill that faculty may need to acquire to maintain well-being and performance when demands from one particular role threaten other roles. Similarly, other participants indicated they could lack focus during teaching when events in other tasks would bother them.

### Teacher actions

Although not part of our initial research question and interview guide, many participants spoke of their actions spontaneously. Our participants clearly described what perceptions or demands prompted them to take action, and why. For example, several participants described including additional content in their lectures beyond what the curriculum asked of them, to challenge themselves or their students. On the other hand, others chose to forego including interactive parts in their lecture as they perceived lectures without interaction to be easier to give.

The changes described were perceived as beneficial changes to the participants, because they made their work easier, better, more fun or more rewarding. It should be noted that these changes may not necessarily have been beneficial for the quality of teaching, as this citation from Agnes shows, when she elaborated on the example of no longer having to mark multiple choice questions: “*I still have to mark the essay questions. And then I naturally make sure I design the essay questions in such a way that they are easy to mark*, *as quickly as possible*”.

On the other hand, Patty, a researcher and educator, clearly described how her curiosity drove her to start undertaking educational research even though the university had not asked her to conduct research. This is probably a beneficial change: “*… And those students went through the first year and then I think*, *how are they performing?* … *I don*’*t know if the* [*board*] *would*’*ve ordered me to research that if I had not initiated it.*”

Broadly, these actions related to alterations in their work environment, their teaching task or their other tasks. Within the teaching task, then, we could make the distinction between changes in content and changes in methods.

## Discussion

In describing their work environment, medical school faculty identify many aspects that directly and indirectly relate to their teaching tasks. They clearly perceive these as a demand or as a resource and may be related to themselves, their tasks and the organization at large. Their descriptions of how this influences their teaching provide insight into a complex and dynamic process in which demands and resources may be interrelated and conditional for other aspects.

Contrasting descriptions from medical school faculty further show the individuality of this process. Similar aspects or elements thereof can be perceived oppositely between individual faculty. Therefore, the perception of the work environment cannot be unambiguously labeled as either a resource or demand.

In describing the interaction between tasks and roles, faculty describe how different tasks and roles interact and how these interactions may be both simultaneously resourceful and demanding. This may be because of the interaction itself, or more indirectly through the relationship between tasks and roles as described above.

Lastly, through medical school faculty’s description of their actions based on the perceived demands and resources, we found that the work environment may be changeable or at least perceived as changeable.

The contribution of our research to the guidance of institutional policy and the design of faculty development is that it provides a view on the work environment as major influence on the well-being and performance of faculty. In our introduction we argued that for supporting work engagement, adding job resources is more important than reducing job demands. DaRosa et al. [42] referred to several barriers to effective teaching—barriers which mirror some of the demands mentioned by our participants. Our study shows that these barriers and demands are connected to certain resources, which provides faculty developers, HRM and boards alike with new leads to support the well-being of their faculty.

In terms of providing resources, research reports on the effect and usefulness of specific career pathways for medical educators [43–46] have shed light on the importance of recognition as well as protected time for teaching. We add to this knowledge by acknowledging the individuality of the influences and complexity of the work environment which could imply that such pathways should have flexible designs. In addition, the beneficial effects of fulfilling multiple tasks and roles are important to consider in designing such tracks.

In relation to faculty development, recent articles have proposed to include a broader focus on faculty development research by including the workplace as an important factor in the development of faculty. [47] How the workplace affects work engagement through the perceived job demands and resources and, through work engagement, the subsequent performance of faculty in the workplace could therefore provide insights in the mechanisms that lead to a supportive workplace. Currently, faculty development often focuses on skill-based interventions [48] or on performance in specific roles [49] but doesn’t necessarily take into account well-being and could benefit from an holistic view on medical school faculty’s performance.

Lastly, several recent articles have provided new insights in identity formation of physicians [50] and faculty [51] in their respective roles. The drivers of identity formation share similarities with drivers of work engagement, such as the importance of recognition, and these studies could benefit from combining their approaches. It is sensible to assume engaged teachers have a stronger teaching identity, although this remains to be studied.

In a recent review of work engagement literature, it was concluded that daily fluctuations of work engagement may be substantial relative to a stable general state [52–54] and that these fluctuations are causally related to changes in daily job resources and personal resources [52].

Based on our results we suggest these changes primarily occur within the task-related resources and that organizational resources are more stable. However, it could also be that the availability of resources for individual teachers changes as working days differ in tasks fulfilled.

Furthermore, earlier research established that a proactive behavior of employees is associated with the preservation of work engagement [52]. This phenomenon is called job crafting [55, 56] and this too has been described as a phenomenon changing from day to day [54]. Some actions described by our participants match this description. Our results suggest that work engagement for the teacher role is primarily maintained by crafting the teaching tasks, rather than crafting the other tasks. However, we did not actively inquire about the perception of control or specifically the perceived autonomy of teachers, which both have been strongly correlated with job crafting [54, 55]. Our results do show that teachers have different needs for autonomy depending on their context. They could need a frame to work within when providing teaching instead of being given full autonomy on form and content.

Both these hypotheses require further research, either through quantification in larger populations or through qualitative inquiry specifically focused on these topics.

It was not our intention to compile an exhaustive list of demands and resources for medical education specifically, but rather to understand how demands and resources interact in the complex environment that is medical education, in which people fulfill multiple roles virtually interchangeably. Still, some demands and resources we found mirror findings in general work engagement literature [23] such as autonomy and performance feedback, and earlier studies into resources experienced by faculty [35].

Findings which have not been described in earlier research are the marked difference between individual perceptions of similar aspects of the work environment. Quantitative research among recently graduated veterinarians has shown that the certain demands and resources may affect well-being to a lesser or greater extent for women than for men [57]. Our study design does not merit comparison between women and men, but at no point during our analyses did any differences become apparent. This is probably due to our small sample, but we suggest that the individuality of the process extends beyond gender. Our practical recommendation would thus be to approach each teacher individually without regarding gender.

In addition, personal *demands* have not yet been studied, while personal *resources* have been studied thoroughly [31, 58–60]. As stated in the introduction, personal resources are ‘aspects of the self that are generally linked to resiliency and refer to the individuals’ sense of their ability to control and impact upon their environment successfully’ [31] It could be argued that personal demands act similar, but oppositely, to personal resources in that they decrease resiliency and sense of control. However, the causal relationship between personal demands, work engagement—and burnout—remains to be studied.

### Strengths, limitations and reflexivity

A strong point of our study is that it is grounded in a well-researched construct, the Work Engagement Model [23], and connects knowledge from the field of organizational psychology with the knowledge from the field of faculty development and management in health care education as described above. We feel that bringing these fields together further strengthens the theoretical foundation of our research.

Our study included teachers from two university teaching hospitals which strengthens our results in terms of transferability to other institutions. However, although the Work Engagement Model has been validated across cultures, nations and professions [23] our study included teachers from a single country, potentially limiting transferability to other cultures on the level of specific demands and resources. We think the described relationships and interactions are transferable.

Furthermore, we purposefully sampled our participants to primarily provide for a wide range of backgrounds and invited teachers with a substantial teaching role. Teachers with limited experience were not included as their teaching role is often limited in time. This could further affect transferability of specific demands and resources. They may perceive job demands and resources differently from more experienced teachers.

In regards to reflexivity in qualitative research, it is important to consider the background of the members of the research team [61]. The authors have backgrounds in medicine, veterinary medicine, medical education, faculty development and health services research. This strengthened the diversity within our discussions. The first author, who conducted all interviews, has a degree in medicine and works as faculty developer which may have affected interviews through being familiar with the language used. This may have left certain statements implicit but also ensured familiarity with the educational systems and participants’ work environment, which allowed the interviewer to act on specific cues provided by the participants.

## Conclusion

Our results suggest that an individualized approach is necessary in order to maintain the well-being of faculty members. Furthermore, while fulfilling the different tasks may lead to role conflict, our results suggest that actively embracing the positive effects can be helpful in lessening the perceived demands that result from role interaction and strengthen the positive effects. Lastly, it is important to acknowledge faculty do take action themselves regardless or even contrarily to what actions are undertaken by heads, boards or institutions at large.
